# A Case of Disseminated Histoplasmosis Presenting As Adrenal Insufficiency With Granulomatous Hepatitis

**DOI:** 10.7759/cureus.45598

**Published:** 2023-09-20

**Authors:** Abaan Khurshid, Zain Satti, Rejath Jose, Chirag Vasa

**Affiliations:** 1 School of Medicine, New York Institute of Technology College of Osteopathic Medicine, Old Westbury, USA; 2 Clinical Sciences, New York Institute of Technology College of Osteopathic Medicine, Old Westbury, USA; 3 Infectious Disease, Mount Sinai Queens Hospital, Astoria, USA

**Keywords:** adrenal glands, histoplasma capsulatum, granulomatous hepatitis, adrenal insufficiency, disseminated histoplasmosis

## Abstract

Histoplasmosis is a fungal infection caused by *Histoplasma capsulatum*. In the United States, histoplasmosis is endemic in the Mississippi River Valley and Ohio. Histoplasmosis is often asymptomatic in immunocompetent individuals, and severe disseminated cases are more often seen in immunosuppressed patients. Disseminated histoplasmosis often affects the reticuloendothelial system, invading specific visceral organs such as the liver, spleen, and pancreas. The current study presents a unique case of disseminated histoplasmosis in a 64-year-old immunocompetent male. The patient's presentation included a 40-lb weight loss over a year, bilateral adrenal nodules, abnormal liver enzymes, and granulomatous hepatitis, which initially raised suspicion of a malignant etiology. An adrenal mass biopsy showed fungal morphology that confirmed an *H. capsulatum *infection. Further history showed that the patient recently traveled to Bangladesh, which is thought to be a region endemic to histoplasmosis. This case is noteworthy because disseminated histoplasmosis rarely affects immunocompetent individuals, and an infectious etiology for adrenal insufficiency is exceedingly rare, especially in the United States. The treatment regimen included a 14-day induction therapy of IV amphotericin B followed by outpatient itraconazole, leading to symptom resolution. This case highlights the need to consider an infectious etiology for adrenal insufficiency, especially among immunocompetent individuals who may be at risk after traveling to endemic areas.

## Introduction

Histoplasmosis is a chronic, infectious disease that most commonly affects the respiratory system. It is the most common fungal infection in the United States and is endemic to Ohio and the Mississippi River Valley. It is caused by *Histoplasma capsulatum*, a dimorphic fungus with a yeast-like structure and septate hyphae at 37°C [1]. Exposure is often due to soil containing bird and bat feces and in those who explore caves or work at specific construction sites where spores from the soil can be inhaled [2,3]. Histoplasmosis in immunocompetent individuals is often asymptomatic or present with minimal symptoms. Individuals with severe or disseminated infections are often immunocompromised patients with organ or stem cell transplants, Human Immunodeficiency Virus, corticosteroids, and immunosuppressive medications. The gold standard for diagnosing histoplasmosis is either a blood culture demonstrating *H. capsulatum* or histopathologically demonstrating the morphologic forms of *H. capsulatum* in affected tissues [2,4].

Adrenal insufficiency is an uncommon complication of disseminated histoplasmosis and can be hard to manage. Primary adrenal insufficiency (PAI) presents when damage to the adrenal glands causes a deficiency in androgens, cortisol, and aldosterone [5]. Symptoms may include fatigue, weight loss, nausea, and orthostasis. Adrenal insufficiency secondary to disseminated histoplasmosis is potentially life-threatening; however, in patients with disseminated histoplasmosis and adrenal involvement, only 7% had clinically evident primary adrenal insufficiency. The rate of adrenal insufficiency is relatively rare in disseminated histoplasmosis because both adrenal glands need to be replaced with necrotizing granulomatous inflammation for a patient to present with overt adrenal insufficiency [1].

This case illustrates the importance of considering all complications of disseminated histoplasmosis when managing patients, including adrenal insufficiency. Other complications of disseminated histoplasmosis include endovascular infection, meningitis, and hepatic portal lymphohistiocytic inflammation, which are notoriously difficult to manage [1]. The current case is unique due to the presentation of a rapid 40-lb weight loss for one year associated with adrenal nodules, mimicking other differentials such as malignancy.

## Case presentation

A 64-year-old male patient with a past medical history of diabetes mellitus and hyperlipidemia presented to the infectious disease clinic for a follow-up visit after being discharged from the hospital. The patient initially sought care at a New York Emergency Room (ER) two months prior to this office visit due to experiencing chills, fever, and night sweats for a duration of three days leading to an admission into the same hospital. The patient lived in Queens, New York at the time of presentation and for most of his life. He had reported a history of a 40-lb weight loss over the past year. He underwent an extensive workup revealing bilateral adrenal nodules, normal plasma metanephrines, and granulomatous hepatitis via liver biopsy at outpatient facilities, 1 month before this presentation at the same facility. The granulomatous hepatitis was of unknown cause at the time, and the patient began oral prednisone 40 milligrams (mg), 10 mg 4x daily orally, as recommended by the hepatologist. Liver enzymes were not elevated during this disease process. Before initiating prednisone use, the patient showed signs of adrenal insufficiency, such as hypotension with a blood pressure of 97/61 mm Hg and 40 lb of weight loss. The month prior, an esophagogastroduodenoscopy and colonoscopy failed to reveal any possible causes for his symptoms. Table 1 shows the workup and findings before the emergency room (ER) visit. 

**Table 1:** Test results prior to emergency room visit.

Test	Result
Computed Tomography of Thorax	Bilateral adrenal masses, left 3.2x2.6x3.3cm, right 1.4x.10cm.
Liver, needle biopsy specimen	Numerous epithelioid granulomas found throughout the lobules. Findings consistent with granulomatous hepatitis

**Figure 1:**
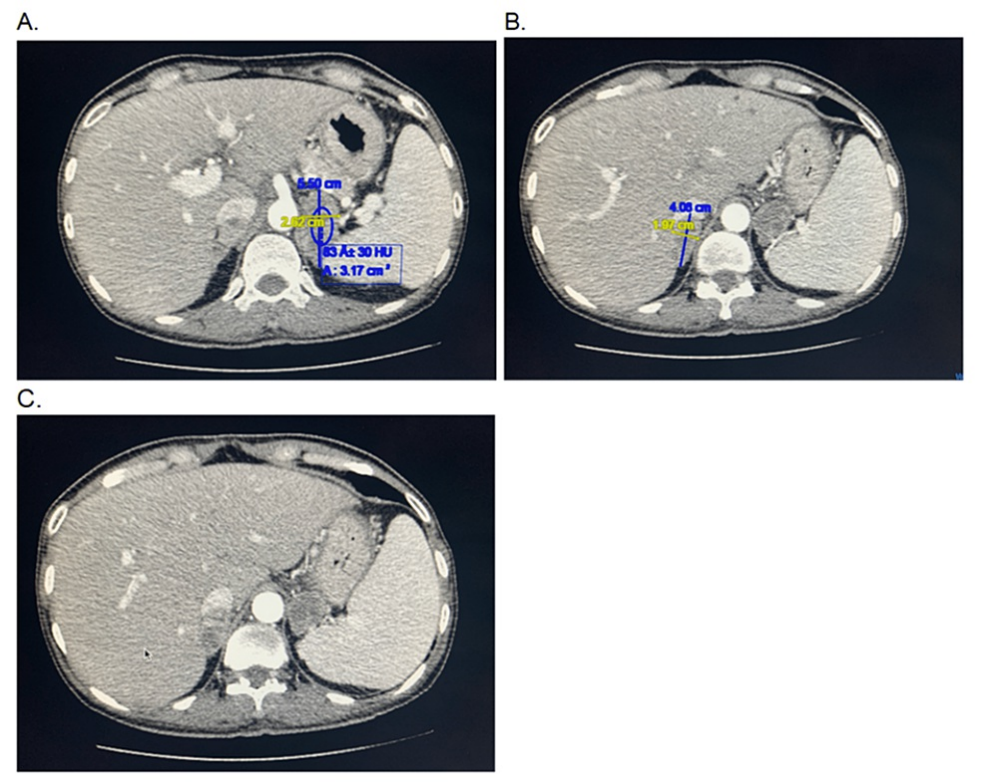
A. Left adrenal mass enlargement (5.5 cm). B. Right adrenal mass enlargement (4 cm). C. Hepatomegaly

The vital signs on presentation to the ER visit included a blood pressure of 99/63 mm Hg, heart rate of 96 beats/min, temperature of 97.5°F, respiratory rate of 18 breaths/min, oxygen saturation of 95% on room air, BMI of 16.91 kg/m^2^.^ ^On presentation to the ER, a head-pelvis Computed Tomography scan was performed, revealing hepatomegaly and hypermetabolic, enlarged adrenal glands measuring up to 5.5 cm in the left and 4 cm in the right with enhancement pattern favoring malignancy rather than adenomas (Figure 1A-1C). Prior workup of pheochromocytoma involving plasma metanephrines was inconsistent with pheochromocytoma, and the patient denied a history of palpitations, diaphoresis, or tachycardia. Of note, an HIV antigen test was negative. Interventional radiology was consulted for adrenal biopsy, as recommended by oncology, after the second pheochromocytoma workup was negative.

**Figure 2:**
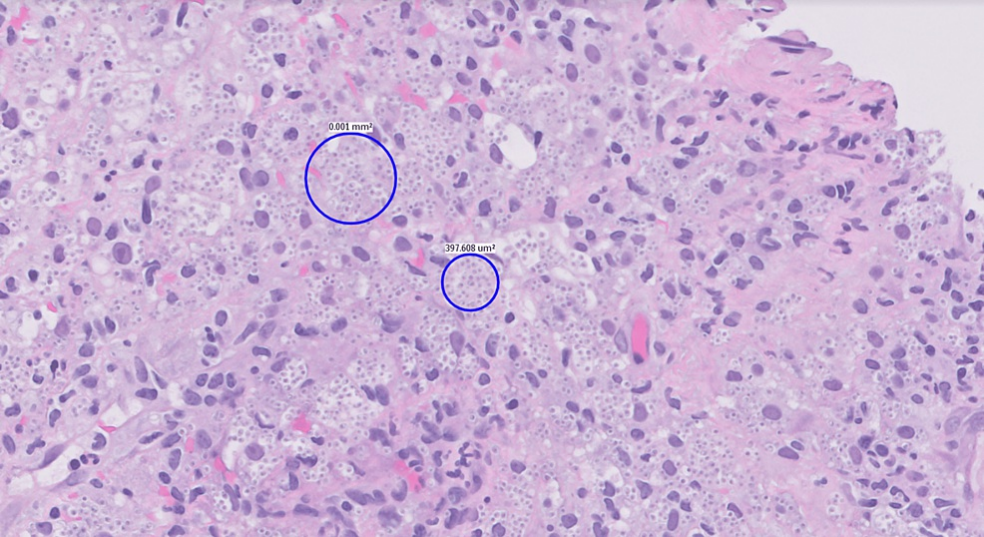
Histopathology showing narrow-based buds and mucin-negative halos.

Adrenal mass biopsy revealed plasma cell-rich chronic inflammation with numerous fungal yeast forms confirmed by Grocott’s methenamine silver stain. The acid-fast bacilli stain was negative for acid-fast organisms, and the mucicarmine stain was also negative. The morphological features of the fungus (narrow-based budding and mucin-negative halo) were consistent with *H. capsulatum* (Figure 2). Histoplasma urine antigen was negative; however, the Beta-D-Glucan assay showed > 500 pg/ml. The patient had a prior history of visiting Arizona and Bangladesh.

After biopsy results demonstrated granulomatous inflammation with numerous fungal yeast forms on Hematoxylin and Eosin stain, the patient was notified and admitted to the hospital to begin a regimen of IV amphotericin B. On admission, the patient was afebrile (97°F) and stable. The patient underwent a 14-day induction therapy of amphotericin B inpatient, which was tolerated well without complications, and subsequently switched to itraconazole for outpatient post-discharge. The patient was concurrently receiving amphotericin B and oral prednisone, which was tapered throughout the hospital course from four doses daily of 10 mg to 2.5 mg. This patient was not using any immunocompromising medications such as steroids during the period of weight loss over the prior year. It is worth noting that the steroid therapy was initiated after the patient experienced weight loss, signifying that the adrenal insufficiency occurred while this patient was immunocompetent.

Upon initiating antifungal treatment, the patient experienced no fever, chills, or night sweats during hospitalization. He was discharged after a 14-day course of amphotericin B, and liver enzymes returned to normal. The patient was discharged on oral itraconazole with a recommended duration of use for 1 year. The patient was instructed to follow up with infectious disease, endocrinology, and hepatology. After amphotericin B induction, the patient’s symptoms of weight loss, fever, chills, and night sweats resolved entirely. This patient was seen by the infectious disease team at the outpatient clinic, where there was no recurrence of adrenal insufficiency. The patient regained weight from 138 pounds to 150 pounds over the course of 6 months after treatments, with no recurrence of symptoms, maintaining itraconazole treatment without using steroid therapy.

## Discussion

Histoplasmosis is typically a self-limiting disease that the human body can clear itself of, if immunocompetent. For unknown reasons, it is more common in males and typically presents with nonspecific symptoms [2,3,6]. A typical differential diagnosis of enlarged adrenal glands includes sarcoidosis, malignancy, hemorrhage, or infections including but not limited to histoplasmosis, tuberculosis, and blastomycosis. Weight loss may point towards a malignancy and a potential metastasis, which should be considered in the differential diagnosis, but it is also important to consider an infectious process. 

While steroids will induce immunosuppression, this patient’s symptoms of adrenal insufficiency began before the steroid regimen was prescribed due to granulomatous hepatitis, another consequence of histoplasmosis. The initiation of steroid treatment worsened this patient’s symptoms and led to the onset of night sweats, fever, and chills, although it is important to note that adrenal insufficiency and granulomatous hepatitis were seen prior to the initiation of steroid use. This implies that dissemination occurred in an immunocompetent host, thus making the case more unique as most reported cases of disseminated histoplasmosis occur in immunocompromised hosts. Regarding diagnosis, histopathological examination of affected adrenal tissue will demonstrate fungal cells that are budding, in yeast form. There may be a visible clear ring of space surrounding spherical yeast forms [3,7]. Inflammation and granulomas may also be present. 

In this case, the patient achieved significant symptom improvement owing to prompt diagnosis, which stands in contrast to an earlier case study by Jagadish et al., where the patient experienced multiorgan failure that led to fatality [3]. Jagadish et al. describe a rare case of disseminated histoplasmosis in an immunocompromised patient that was severe and presented after an organ transplant, where the patient eventually developed poor mentation and eventually passed away. The outcomes for disseminated histoplasmosis may vary and can potentially be related to the time from disease onset to initiation of treatment. Therefore, this case highlights an important teaching point regarding the clinical consideration of histoplasmosis in patients with suggestive travel history, adrenal insufficiency, and systemic symptoms. Furthermore, imaging suggestive of adrenal abnormalities should prompt a timely biopsy for a definitive diagnosis so that appropriate treatment may be started.

In patients with disseminated histoplasmosis who are experiencing severe symptoms such as weight loss and night sweats, inpatient care is recommended with intravenous amphotericin B over the course of 1-2 weeks and subsequently outpatient follow-up with itraconazole daily over 1 year, as was done in this patient [8]. Occasionally, persistent steroid use may be indicated if adrenal insufficiency persists. In this patient, steroids were tapered and discontinued after discharge due to adrenal recovery. With proper antifungal treatment, this rare disease process that is potentially fatal may be curable with complete resolution, as described in this case.

A case by Madhavan et al. involved disseminated histoplasmosis with a patient suffering from adrenal insufficiency who eventually required long-term glucocorticoid supplementation after antifungal treatment due to persistent adrenal insufficiency [9]. Even after the successful treatment of histoplasmosis using antifungal agents, the patient continued to exhibit symptoms of adrenal insufficiency. Due to this inability to regain adrenal function, the patient had to be placed on long-term steroid supplementation. Typically, once histoplasmosis is resolved, the affected organs heal and regain their ability to produce necessary glucocorticoids. However, this particular case underscored a rare scenario where, despite the resolution of the disseminated histoplasmosis, the adrenal glands' loss of function was irreversible. 

Such instances demonstrate the importance of monitoring and follow-up in patients with disseminated histoplasmosis, regardless of infectious resolution, to ensure that any long-term complications may be managed appropriately. Our patient did not display this loss of function when seen in the infectious disease clinic after disease resolution. The presence of adrenal insufficiency emphasizes that this case is distinct in its presentation of histoplasmosis as it is very rare for patients to present with clinically evident primary adrenal insufficiency.

## Conclusions

Disseminated histoplasmosis that affects bilateral adrenal glands as well as the liver is rare, with literature citing few cases a year. It is much more common in immunocompromised hosts, albeit still rare. Severely symptomatic cases in immunocompetent individuals with disseminated histoplasmosis are extremely rare, as seen in this case. This patient did not have underlying HIV or other immunocompromising disease, yet still suffered dissemination to the bilateral adrenal glands and liver. This case aims to highlight that although there's an established relationship between an immunocompromised state and disseminated histoplasmosis, it should be ruled out in an immunocompetent patient with a suggestive history. Prompt diagnosis of the disease is associated with improved outcomes, so the knowledge of different disease manifestations is essential for a timely diagnosis. Therefore, our report aims to raise awareness and enhance education among clinicians and practitioners regarding this disease process and its potential occurrence among immunocompetent individuals.
